# Ex Vivo Expansion and Drug Sensitivity Profiling of Circulating Tumor Cells from Patients with Small Cell Lung Cancer

**DOI:** 10.3390/cancers12113394

**Published:** 2020-11-16

**Authors:** Hsin-Lun Lee, Jeng-Fong Chiou, Peng-Yuan Wang, Long-Sheng Lu, Chia-Ning Shen, Han-Lin Hsu, Thierry Burnouf, Lai-Lei Ting, Pai-Chien Chou, Chi-Li Chung, Kai-Ling Lee, Her-Shyong Shiah, Yen-Lin Liu, Yin-Ju Chen

**Affiliations:** 1 Department of Radiation Oncology, Taipei Medical University Hospital, Taipei 11031, Taiwan; b001089024@tmu.edu.tw (H.-L.L.); solomanc@tmu.edu.tw (J.-F.C.); 123007@h.tmu.edu.tw (L.-S.L.); 971010@h.tmu.edu.tw (L.-L.T.); 2 Taipei Cancer Center, Taipei Medical University, Taipei 11031, Taiwan; ylliu@tmu.edu.tw; 3 Department of Radiology, School of Medicine, College of Medicine, Taipei Medical University, Taipei 11031, Taiwan; 4 TMU Research Center of Cancer Translational Medicine, Taipei Medical University, Taipei 11031, Taiwan; 5 Shenzhen Key Laboratory of Biomimetic Materials and Cellular Immunomodulation, Shenzhen Institute of Advanced Technology, Chinese Academy of Sciences, Shenzhen 518055, Guangdong, China; pengyuanwang@swin.edu.au; 6 Department of Chemistry and Biotechnology, Swinburne University of Technology, Hawthorn, VIC 3122, Australia; 7 Graduate Institute of Biomedical Materials and Tissue Engineering, College of Biomedical Engineering, Taipei Medical University, Taipei 11031, Taiwan; thierry@tmu.edu.tw; 8 International Ph.D. Program in Biomedical Engineering, College of Biomedical Engineering, Taipei Medical University, Taipei 11031, Taiwan; 9 Genomics Research Center, Academia Sinica, Taipei 11529, Taiwan; cnshen@gate.sinica.edu.tw; 10 Graduate Institute of Clinical Medicine, College of Medicine, Taipei Medical University, Taipei 11031, Taiwan; 11 Division of Pulmonary Medicine, Department of Internal Medicine, Wan Fang Hospital, Taipei Medical University, Taipei 11600, Taiwan; 94401@w.tmu.edu.tw; 12 School of Respiratory Therapy, College of Medicine, Taipei Medical University, Taipei 11031, Taiwan; 13 Division of Pulmonary Medicine, Department of Internal Medicine, Taipei Medical University Hospital, Taipei 11031, Taiwan; 171010@h.tmu.edu.tw (P.-C.C.); clchung@tmu.edu.tw (C.-L.C.); d118107005@tmu.edu.tw (K.-L.L.); 14 Department of Internal Medicine, School of Medicine, College of Medicine, Taipei Medical University, Taipei 11031, Taiwan; 15 Division of Thoracic Medicine, Department of Internal Medicine, School of Medicine and School of Respiratory Therapy, College of Medicine, Taipei Medical University, Taipei 11031, Taiwan; 16 Graduate Institute of Cancer Biology and Drug Discovery, College of Medical Science and Technology, Taipei Medical University, Taipei 11031, Taiwan; hss121010@tmu.edu.tw; 17 Division of Hematology and Oncology, Department of Internal Medicine, Taipei Medical University Hospital, Taipei Medical University, Taipei 11031, Taiwan; 18 Department of Pediatrics, School of Medicine, College of Medicine, Taipei Medical University, Taipei 11031, Taiwan; 19 Department of Pediatrics, Taipei Medical University Hospital, Taipei 11031, Taiwan

**Keywords:** small cell lung cancer, circulating tumor cells, primary cell culture, liquid biopsy, personalized medicine

## Abstract

**Simple Summary:**

Small cell lung cancer (SCLC) is an overly aggressive cancer characterized by rapid growth, early metastatic spread, and consequently reducing overall survival. As cancer manifestations can differ uniquely between various types, the rapid proliferation of circulating tumor cells (CTC) originating from SCLC was an adequate sample resource to aid the headway in our drug screening technique. With biomarker detection of liquid biopsy as an emerging tool to assist decision-making in personalized cancer pharmacotherapy. In this study, we developed a rapid and reproducible system for preclinical drug testing via the use of a unique CTCs expansion protocol. The expanded CTCs from SCLC formed multiple types of tumorsphere structures and expressed SCLC-specific tumor markers. The drug sensitivity assessment gathered from in vitro expansion of CTCs was able to generate positive clinical therapeutic outcomes. Thus, SCLC patient-derived CTC spheroids are a useful resource for biomarker development and drug sensitivity assessment providing “real-world” therapeutic response circumstances.

**Abstract:**

Small cell lung cancer (SCLC) represents one of the most aggressive malignancies among cancer types. Not only tumor sample availability is limited, but also the ability for tumor cells to rapidly acquire drug resistance are the rate-limiting bottlenecks for overall survival in current clinical settings. A liquid biopsy capable of capturing and enriching circulating tumor cells (CTCs), together with the possibility of drug screening, is a promising solution. Here, we illustrate the development of a highly efficient ex vivo CTC expansion system based on binary colloidal crystals substrate. Clinical samples were enrolled from 22 patients with SCLC in the study. The CTCs were enriched and expanded from the collected peripheral blood samples. Expanded cells were analyzed for protein expression and observed for drug sensitivity with the use of immunofluorescence and ATP titer evaluation, respectively. Successful CTC spheroid proliferation was established after 4 weeks within 82% of all the collected peripheral blood samples from enrolled patients. Upon immunofluorescence analysis, the enriched cells showed positive markers for EpCAM, TTF-1, synaptophysin and negative for CD45. Additionally, the expanded CTCs demonstrated marked heterogeneity in the expression of E-cadherin and N-cadherin. In a preliminary case series, the drug sensitivity of patient-derived CTC to cisplatin and etoposide was studied to see the correlation with the corresponding therapeutic outcome. In conclusion, our study demonstrates that it is possible to efficiently expand CTCs from SCLC within a clinically relevant time frame; the biomarker information generated from enriched CTCs can assist the selection of effective drugs and improve disease outcome.

## 1. Introduction

Small cell lung cancer (SCLC) represents 13% of all newly diagnosed lung cancer cases, and it is one of the most aggressive malignancies with the early development of metastases. The prognosis of SCLC remains dismal, with a median overall survival of 10 months and a 5-year survival rate of 5% even in the early stages of the disease [1,2]. The standard first-line treatment of SCLC consists of a platinum doublet: cisplatin or carboplatin and paired with etoposide. Although initial response to chemotherapy is common, acquired resistance with high relapse rates within one year is the major cause of treatment failure [2,3]. The scarcity of tumor tissue available for research and the high necrotic content of small tumor biopsy further limited the establishment of research models to unravel SCLC biology and explore druggable targets. As a result, overall survival has yet to see a substantial improvement during the past four decades [2,3,4]. Having access to patient-derived tumor materials for multimodalities research may be the first step towards the new therapeutic paradigm.

Circulating tumor cells (CTCs) analysis is a promising new diagnostic field for estimating the risk of metastatic relapse and metastatic progression in patients with cancer [5,6]. Among biomarkers, CTCs are a convenient, sensitive and biologically informative option. The prognostic value of CTCs has been reproducibly validated in several high-quality clinical trials. Accumulating evidence shows that CTCs are abundant in SCLC patients, and CTC enumeration has been demonstrated as a prognostic marker in patients receiving chemotherapy [7,8,9]. Furthermore, the presence of apoptotic CTCs or CTC clusters is associated with a worse survival outcome [7]. In addition to enumeration, different approaches for CTC analysis is also promising in personalized cancer medicine [10,11]. For example, CTCs proliferation has been reported to be a biomarker for drug sensitivity profiling. Yu et al. have demonstrated in a proof-of-concept study that combining genomic sequencing, CTC culture and xenograft, the optimal combination of oncological drugs can be identified [10]. However, it was difficult to culture CTC in a clinically relevant time frame reproducibly. Growth factor formulation, hypoxia and gentle cell enrichment technology have been proposed as a possible solution in the setting of breast, colon and lung cancers [10,12], and the yield rate of successful CTC expansion has been between 20 and 30% [10,12,13,14,15]. Among various efforts, CTC-derived cell lines had been established and functionally characterized by patients with SCLC [16]. From the research, large cluster formation and epithelial-mesenchymal transition appear to be novel drug resistance mechanisms. Although still in its infancy, the application of CTC expansion shows a great potential to obtain patient-derived materials and facilitate molecular and cellular understanding of SCLC.

In this study, we aimed to establish an efficient, reproducible protocol for ex vivo expansion of CTC and its phenotype characterization and to show its potential application for improved personalized cancer therapy.

## 2. Results

### 2.1. Reproducible CTC Ex Vivo Expansion in SCLC

**Figure 1:**
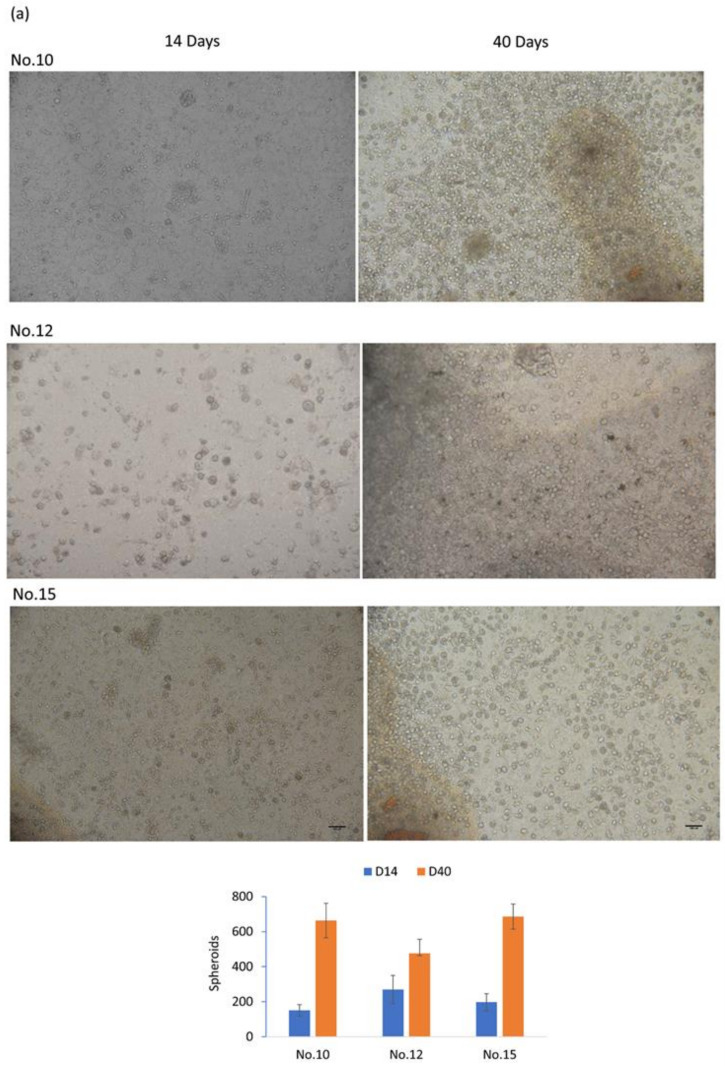

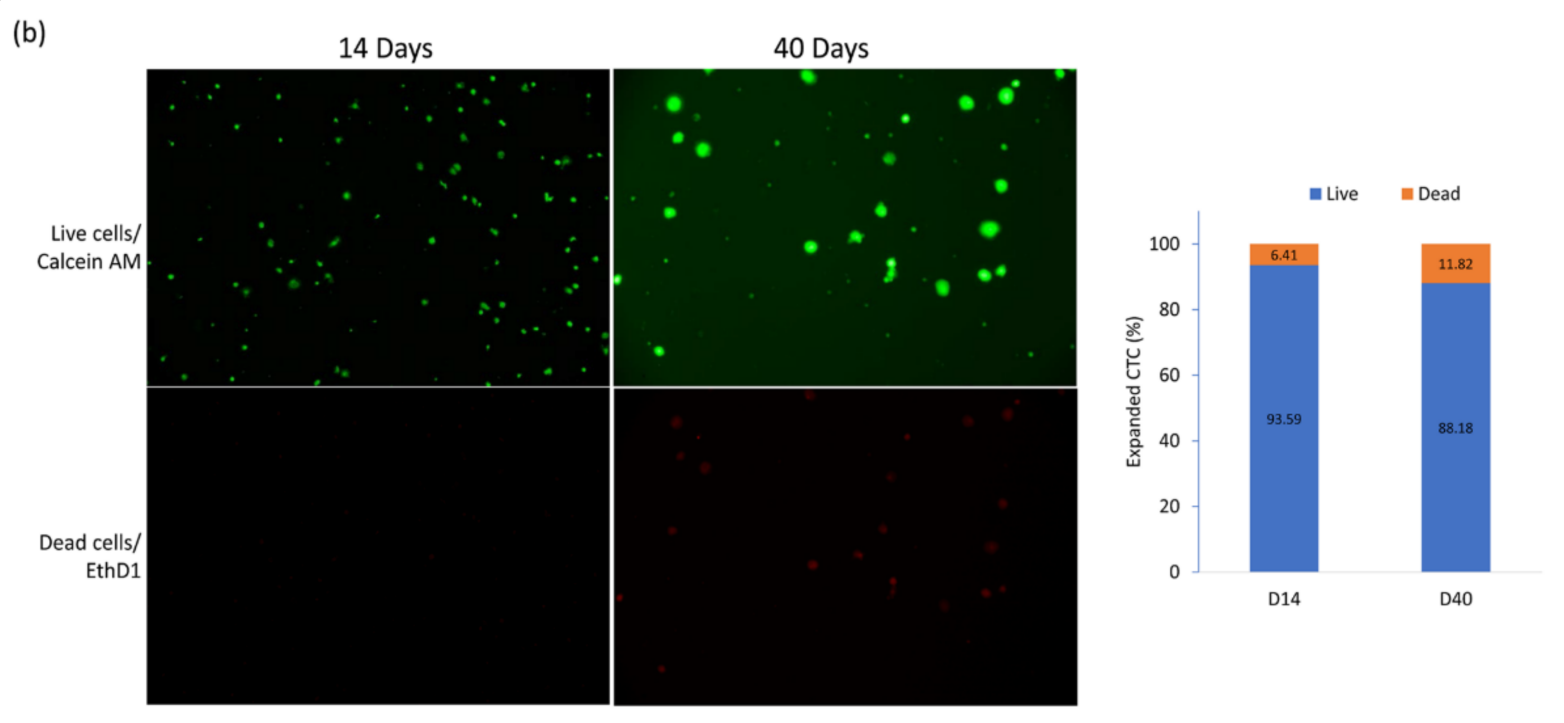
Ex vivo expansion of CTCs from small cell lung cancer (SCLC) patients. (**a**) Light microscopic images (upper panel) and quantitative analysis of spheroid numbers (lower panel) of the expanded CTCs 14 (left) and 40 (right) days. (**b**) The viability of expanded CTCs. On day 14 and day 40, the expanded CTCs were stained with a LIVE/DEAD staining kit. Green and red colors, respectively, indicate live and dead cells. The bar graph presents the percentage of live and dead cells in expanded CTCs.

**Figure 2:**
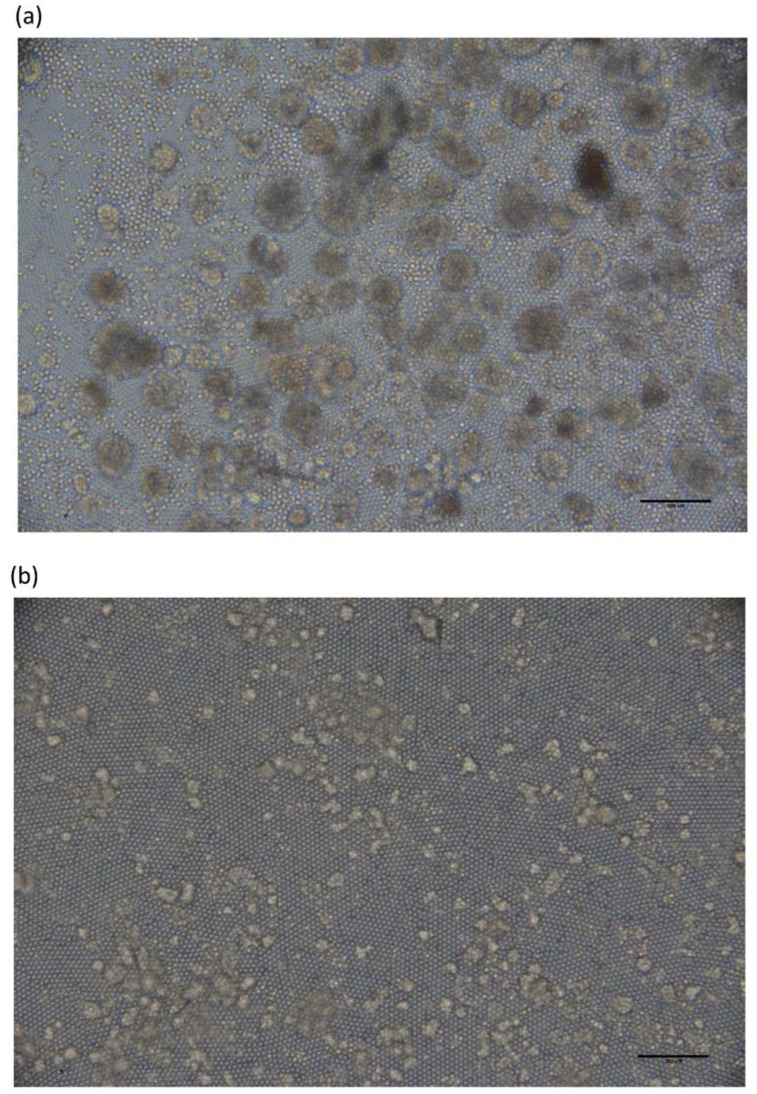

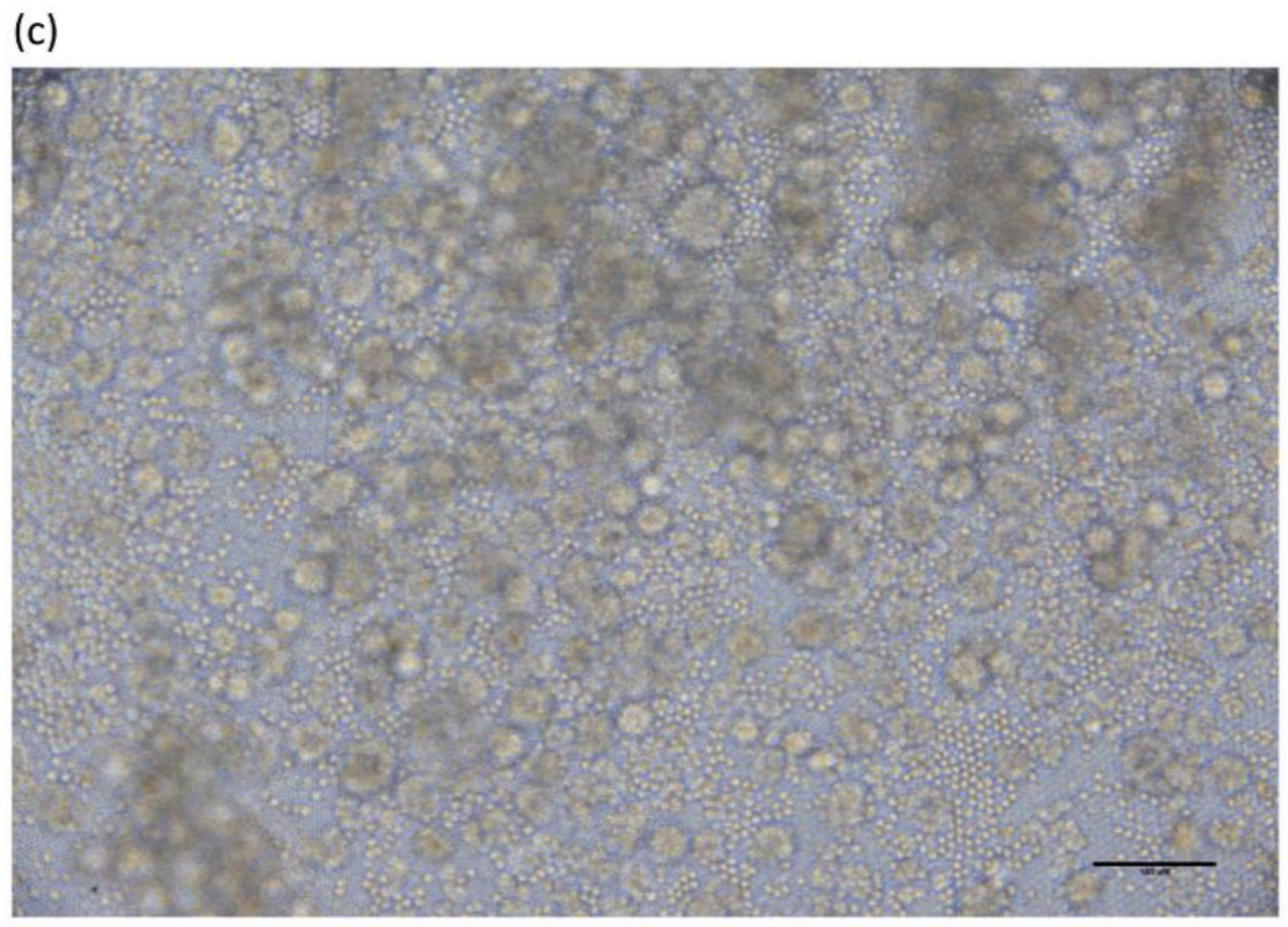
Bright-field images of CTC-derived spheroid phenotypes. Representative examples of expanded CTCs morphology are shown, (**a**) large cohesive CTC spheroids (left), (**b**) small regular and irregular cohesive CTC spheroids (middle) and (**c**) discohesive CTC spheroids (right). Scale bar indicates 100 μm.

The research protocol was approved by the institutional human research board. Peripheral blood samples were collected from patients with pathologically confirmed SCLC. The demographic profile of enrolled patients is shown in Table 1. The peripheral blood mononuclear cells (PBMC) were prepared with Ficoll-Paque centrifugation. CTCs were enriched with antibody cocktail and depletion of residual red blood cells. The enriched CTCs were culture in home-made culture plates coated with self-assembling binary colloidal crystals. CTC spheroids were observed after 14 days and continued to enlarge. The CTC colony numbers increased and formed CTC spheroids with expanded size over time. Representative images and quantified spheroids of CTC derived from SCLC at day 14 and day 40 are shown in Figure 1a. The viability and morphology of expanded CTCs were examined by LIVE/DEAD staining, and results showed that 93.59% and 88.18% CTCs were viable at 14 and 40 days (Figure 1b). The morphology of CTC-derived spheroids differed significantly, as observed by bright-field microscopy. Typically, the morphological construct consists of three different presentable types: large-sized, cohesive round-shaped spheroids (Figure 2a), small-sized cohesive irregular or round spheroids (Figure 2b) and discohesive “grape-like” spheroids (Figure 2c). In this study, we successfully expanded CTCs from 18 of the 22 SCLC patients, with an expansion success rate of 81.8%. By analyzing the results of our study, we revealed a highly effective and reproducible ex vivo CTC expansion system for SCLC.

### 2.2. Immunofluorescence-Based Identification of Expanded CTCs

**Figure 3:**
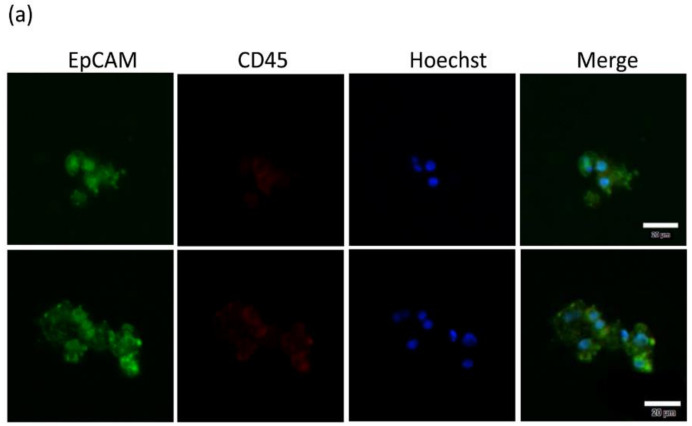

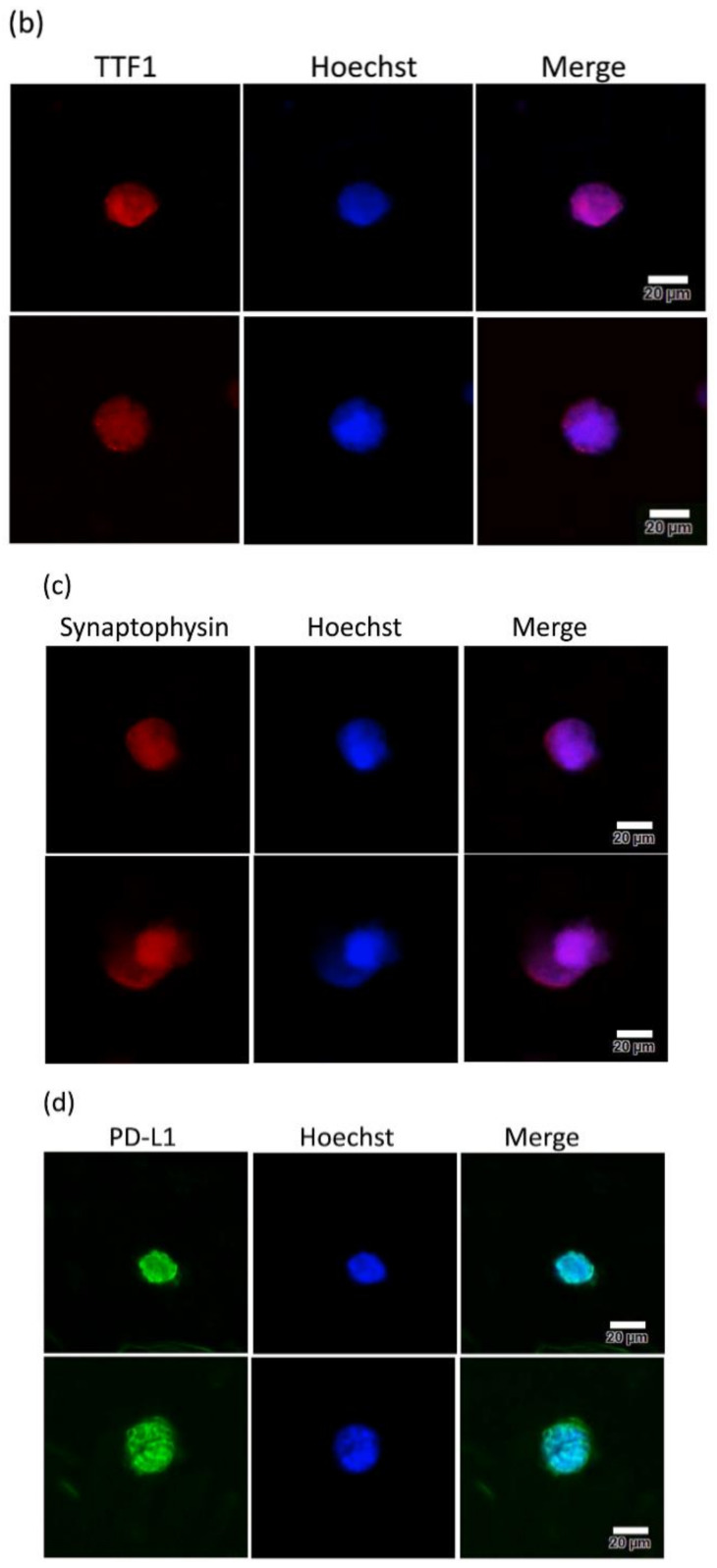
Molecular characterization of expanded CTCs from SCLC patients. Immunofluorescence staining revealed (**a**) EpCAM+ (green)/CD45- (red)/Hoechst+ (blue) cultured CTCs. Prevalent SCLC biomarkers of (**b**) TTF-1 and (**c**) synaptophysin were used to confirm that expanded CTCs derived from lung tissues. (**d**) PD-L1 expression in SCLC. Nuclei of cells were stained with Hoechst 33342 (blue). Scale bar indicates 20 μm. Images are representative of two independent SCLC patient samples.

In this study, expanded CTCs were defined as EpCAM and Hoechst 33342-positive and CD45-negative cells by immunofluorescence staining and visualized by fluorescence microscopy, as shown in Figure 3a. Consistent with the fact that SCLC is a malignant neuroendocrine tumor of lung origin, the expanded CTCs were positively stained for neuroendocrine marker synaptophysin and lung tumor marker TTF-1 (Figure 3b,c). Furthermore, we detected PD-L1 protein expression in the enriched CTCs (Figure 3d).

### 2.3. Epithelial and Mesenchymal Heterogeneity of Expanded CTCs

**Figure 4:**
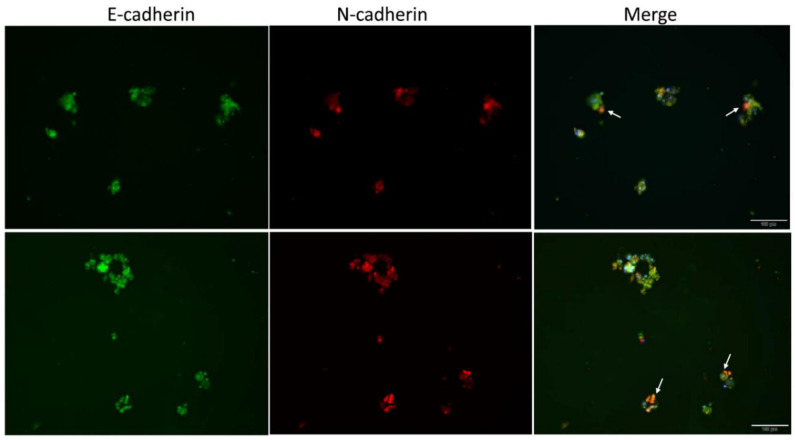
The heterogeneous composition of expanded CTC. Co-staining for E-cadherin (green) and N-cadherin (red) in expanded CTC spheroids reveals the presence of marked heterogeneity for expressions of epithelial and mesenchymal markers. Arrows indicate high levels of N-cadherin.

Recent evidence has revealed that CTCs are heterogeneous and can be sub-classified as circulating mesenchymal cells (CMCs), circulating endothelial cells (CECs) [17]. Unlike other enrichment processes, our selection system did not implement the use of epithelial marker EpCAM as a positive selection for CTCs; thus, we expected heterogeneity to exist in the expanded CTC spheroids consisting of both epithelial and mesenchymal sub-classifications. This, in turn, represented more closely CTC heterogeneity in vivo. To determine this concept, we analyzed the expression levels of the epithelial marker, E-cadherin and the mesenchymal marker, N-cadherin, among the CTC spheroids within our research. The immunostaining data of CTC spheroids revealed the presence of mixed differential expressions within samples of the same patient. In the immunostaining microscopic images, some CTCs solely displayed upregulated E-cadherin, but also some co-expressed N-cadherin or N-cadherin alone (Figure 4). Thus, we observed and classified CTCs from SCLC into the following three subpopulations: epithelial, mesenchymal and intermediated.

### 2.4. Drug Sensitivity Profile of Expanded CTCs Correlates to Treatment Outcome

Yu et al. successfully generated CTC cell lines from breast cancer patients in the application of predicting drug sensitivity [10]. We hypothesized the paradigm is also applicable for SCLC; thus, we conducted experiments to explore the feasibility of using expanded CTC from diagnosed SCLC patients for in vitro drug sensitivity tests.

**Figure 5:**
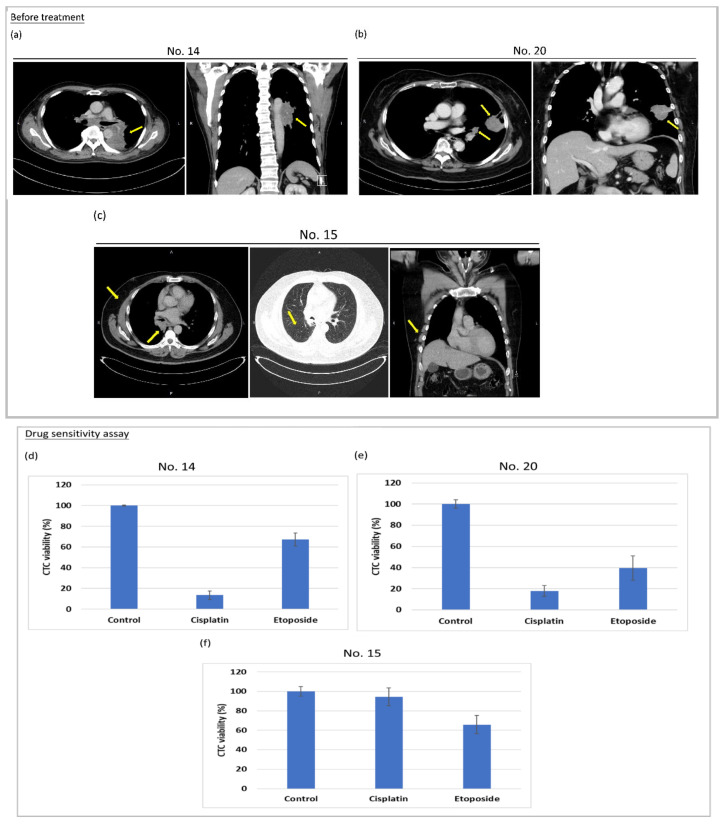

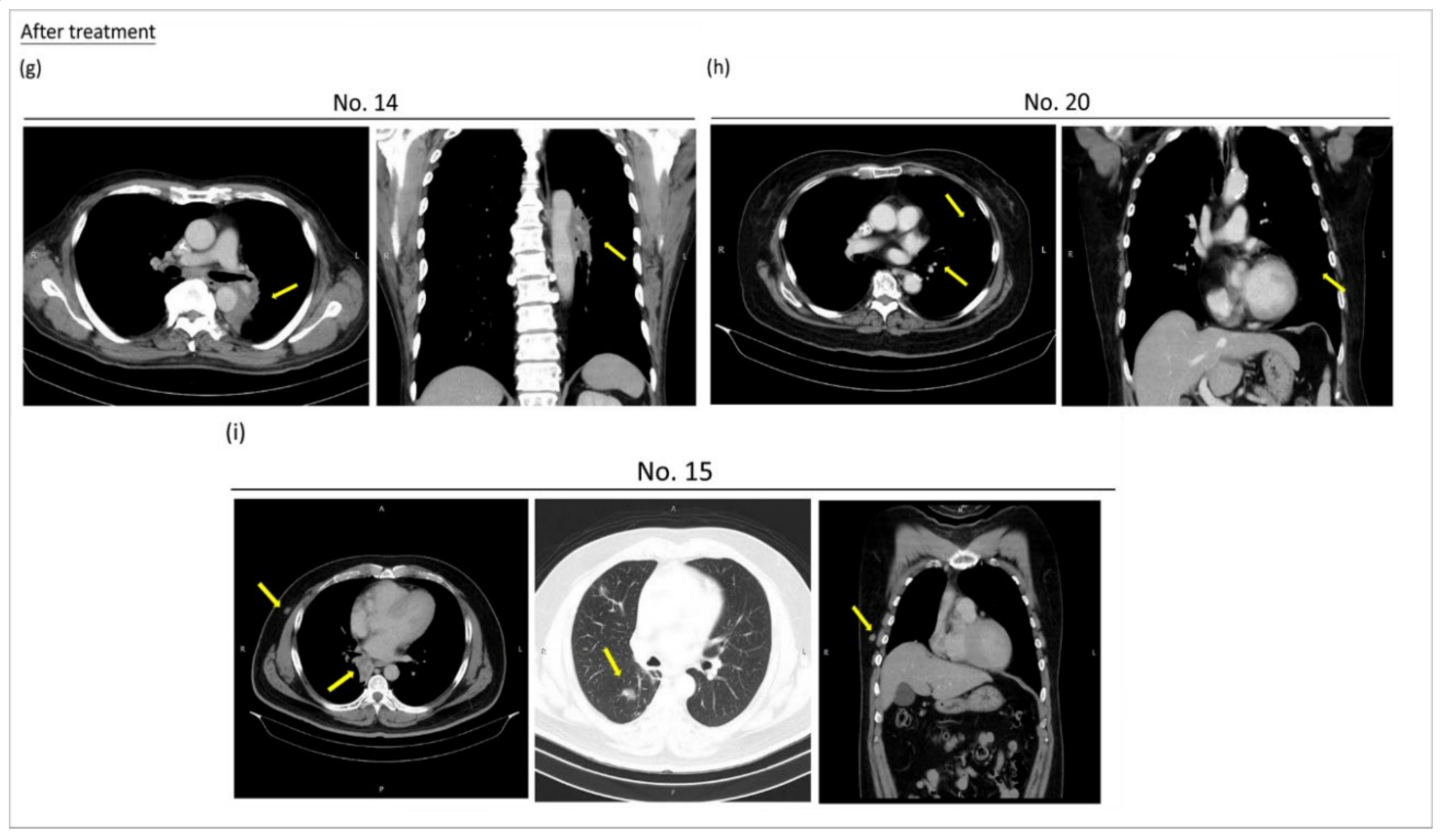
Expanded CTCs recapitulate the clinical response. SCLC patients’ response to standard of care treatment (cisplatin/etoposide) was evaluated by contrast-enhanced CT images before (**a**–**c**) and after (**g**–**i**) treatment. Arrows indicate tumor lesions. Before treatment, blood samples were collected to amplify CTCs and expanded CTCs were plated in 96-well plates for drug sensitivity assay. (**d**–**f**) Cell viability was detected by CellTiter Glo, and results were normalized to the untreated control group. Patients (No. 14 and No. 20) were sensitive to cisplatin/etoposide or (No. 15) resistant to cisplatin/etoposide as evaluated by a CTC drug sensitivity assay and clinical CT images.

Blood samples were collected before the administration of chemotherapy, and from it, the expanded CTCs were submitted for sensitivity assay towards cisplatin and etoposide. Enriched and expanded CTCs were plated, then cisplatin and etoposide were added to the culture medium. After six days, cell viability was examined using CellTiter-Glo^®^ Luminescent Cell Viability Assay. In the meanwhile, donor patients which partook in the liquid biopsy for CTCs expansion mentioned above received standard care for treating SCLC. This was conducted with the administration of cisplatin/etoposide. After three months of standard care, the post-treatment clinical response of these patients was evaluated. Clinical responses and follow-up imaging studies correlated with the results from the drug sensitivity test performed on the expanded CTC in vitro. Figure 5 illustrates the concordant results between drug sensitivity prediction of expanded CTC and that of clinical responses among three patients. Patients 14, 15, 20 were diagnosed with limited-stage cT4N3M0, extensive-stage cT4N3M1b and extensive-stage cT2aN1M1a, respectively. Evaluation with CT image determined the tumor size at the time of initial diagnosis (Figure 5a–c), and then patients received chemotherapy with cisplatin and etoposide at 21–28 days interval in concordance with standard care.

Further examination of the drug sensitivity profile report showed the expanded CTCs from patient No. 14 and No. 20 were displaying sensitivity towards cisplatin and etoposide. Among inspection, the treated cells were displaying significantly reduced viability (Figure 5d,e). In contrast, no significant cytotoxicity action was observed within the expanded CTC from patient No. 15 when exposed to cisplatin and etoposide (Figure 5f).

The initial clinical response from the standard of care was evaluated by chest computed tomography after two cycles of chemotherapy. Patient No. 14 showed mild regression of the primary tumor, and patient No. 20 showed a significant response with dramatic shrinkage of the left upper lung mass (Figure 5g,h). Distinctly, after two cycles of chemotherapy, patient No. 15 revealed mild enlargement of the primary tumor and developed new pulmonary and subcutaneous metastases on the right chest wall (Figure 5i). The above comparison between expanded CTC drug sensitivity assay and patient clinical response was concordant. The results give rise to the feasibility of predicting treatment outcome in individual patients by employing CTC drug sensitivity assay.

## 3. Discussion

Recent studies have revealed that CTCs could serve as a promising blood-based biomarker, and CTC enumeration could predict cancer progression and survival prognosis in various cancer types [5,18,19,20]. Additionally, CTCs are a critical component of liquid biopsies, which have many advantages when compared to tissue biopsies; they are less invasive, more accessible, allow repeated sample collection and serve as real-time detections to reflect current tumor dynamics [10,15]. Moreover, some reports have also pointed out the advantages and opportunities for CTCs use in research and clinical applications, such as molecular characterization to provide comprehensive tumor biological information, illustrating tumor heterogeneity and allowing functional in vitro/in vivo assays. By acquiring these rarely accessible data, we could potentially influence and change treatment modalities. However, until now, the rarity and fragility of CTCs are technological limitations that restrict further exploration and characterization of CTCs [15,21].

Increasing numbers of studies have focused on different strategies for large-scale amplification of CTCs from rare numbers within patients’ samples. For example, the CTC-iChip system, which negatively depleted normal blood cells and then enriched CTCs that are incubated in serum-free media supplemented with growth factors under hypoxic conditions. Other attempts include establishing CTC cell lines from 16% (6 of 36) ER-positive metastatic breast cancer patients [11]. Nevertheless, the fabrication of CTC culture devices is complicated and time-consuming. Studies have explored Co-culturing techniques that have been used to cultivate stem cells, which were adopted for CTC expansion on the basis that feeder cells provide biological signals that may promote CTC proliferation [22,23]. In this context, microfluidics was combined with a fibroblast co-culture system to generate a CTC expansion system for lung cancer samples, and magnetic force-based fibroblast feeder ex vivo CTC culture was proven in an animal-derived CTC model [23]. However, distinguishing pure CTCs and fibroblasts for functional analyses remains a challenge with this strategy.

Although the above-mentioned methodologies provide ex vivo CTC expansion, the complexity of isolation and expansion is still encumbered with low-efficiency and effusively time-consuming, therefore limiting CTC in many clinical applications. In this study, we developed an effective and reproducible ex vivo system for CTC expansion from SCLC patients. By implementing a biomimetic material called binary colloidal crystal (BCC) monolayers, building a complex topographic surface structure that displayed the capacity for expanding embryonic stem cells (ESCs) and inducing pluripotent stem cells (iPSCs) and mesenchymal stem cells (MSCs) [24,25,26,27,28]. With the use of BCC as a bio-mimetic system, an optimized surface can be established to avoid harsh damages to these rare and fragile CTCs and aid the expansion of CTCs ex vivo. Cancer cells are immortal cells sustained by enabling biological proliferative signaling. With the BCC substrate, we create a contact surface that is capable of inducing CTC proliferation and self-renewal.

In our biomimetic environment, we found that CTC spheroids formed and continued to grow for two-weeks after seeding. Samples from SCLC patients had an expansion success rate of 81% (18/22 cases). The successfully expanded CTCs expressed standard CTC markers being positive for EpCAM/pan-cytokeratin and negative for CD45. Furthermore, the tissue-specific markers of TTF-1 and synaptophysin demonstrated expanded CTCs were originated from lung epithelial and not hematopoietic cells (Figure 3). As shown in Figure 2, almost the entire population of expanded CTCs appeared as spheroids, which was also reported in expanded CTCs from SCLC, breast and bladder cancer [16,29,30]. Some factors may explain these results: Among CTCs, small populations of initiating cancer stem cells (CSCs) or cancer initiation cells (CICs) possess properties of stem cell self-renewal, differentiation, and create the heterogeneous lineages of cancer cells. CSCs possess the greatest invasive and metastatic capacity, in turn contributing to distal tumor seeding, clonal expansion and treatment resistance [31,32]. The sphere-formation assay is a widely used method to assess CSC potential [33,34], and spherical formation develops from the proliferation of a single cancer stem/progenitor cell. Therefore, most expanded CTCs may exhibit cancer stemness properties and tend to be more aggressive [34].

Evidence from immunophenotyping and molecular profiling suggests, like primary tumors and metastatic lesions, CTCs show substantial intratumoral heterogeneity (ITH) with the accumulation of mutations, thus resulting in subclones with different levels of fitness [35,36]. It is also reported, functional ITH contributes to tumor biology, tumor invasion, therapeutic resistance and relapse [35,36]. Studies also show CTCs have been classified into circulating mesenchymal cells (CMCs), putative circulating stem cells (CSCs) and circulating endothelial cells (CECs) by different markers [17]. Among these, several reports have demonstrated that CTCs exhibit dynamic changes in epithelial (E) and mesenchymal (M) compositions in various cancer types, and the E/M ratio can distinguish patients who clinically respond to treatment [16,29,37]. As mentioned above, expanded CTCs showed that tumor spheroids might be enriched from a subpopulation of CSCs. In metastatic breast cancer models, CTCs were found to be highly enriched among cells displaying the CSC phenotype [37,38]. Recently, a study identified a subpopulation of CTCs as metastasis-initiating cells from breast cancer patients who revealed high expression levels of stem cell markers, including CD44. The detection of hepatocyte growth factor receptors MET, and CD47 was associated with increased metastasis and reduced overall survival [39]. The same observations were also reported in colorectal cancer and hepatocellular carcinoma [39]. The different subpopulations of cells found in our ex vivo expansion system displayed prevalence of phenotypic plasticity, thus the presence of epithelial-to-mesenchymal transition EMT (Figure 4), spheroid formation (Figure 1 and Figure 2), and programmed death-ligand 1 (PD-L1) (Figure 3) was apparent. These findings reflect the diversity of CTCs and dynamic changes in CTCs. Especially the presence of PD-L1 on expanded CTC has the potential to be further investigated on its correlation to response to immune checkpoint blockade, which is an emerging effective therapy for SCLC [40]. Evidently, our system provides a useful tool for investigating the clinical impacts of cellular heterogeneity present within CTC populations.

SCLC is a “recalcitrant” cancer based on its high lethality; in addition to chemotherapy, the management of SCLC has not changed over the last four decades. The current systemic treatment of SCLC relies on cytotoxic therapies, with first-line regimens consisting of a platinum doublet with cisplatin or carboplatin, generally paired with the topoisomerase inhibitor, etoposide [4]. Based on the time needed to complete first-line chemotherapy, patients may be classified as resistant or sensitive as evaluated by clinical imaging. Currently, there are no validated markers for predicting the response of SCLC to guide decisions regarding the continuation of chemotherapy, a change of regimens or administering of other targeted therapies. Recently, drug development has shifted to the discovery of druggable targets based on tumor biological functions of SCLC [41], theses include aurora kinase inhibitor targeting MYC amplification [42] as an enhancer of zeste homolog 2 (EZH2) inhibitor [43], poly [ADP-ribose] polymerase (PARP) inhibitors and Wee1 inhibitors targeting homologous repair machinery and DNA damage response pathway [41,42,43,44,45]. However, a lack of sufficient tissue samples to analyze for predictive biomarkers is a major bottleneck for drug development and personalized treatment decisions. Expanded CTC has the potential to provide real-time liquid biopsy materials for large scale sequencing and subsequent therapeutic biomarker discovery. In the current investigation, however, genetic sequencing is limited as the quantity of cells after drug sensitivity profiling was not sufficient for quality DNA preparation as per next-generation sequencing standards.

Generally, it takes 1 to 2 months of diagnostic workup and three months of drug treatment for a course of anticancer therapy. Within this time frame, it is not realistic to generate CTC lines or patient-derived xenograft mouse models to inform clinical decisions for personalized treatment. Herein, we established a rapid, reproducible CTC-based model for preclinical drug testing and displayed a close correlation with the clinical treatment response (Figure 5), which can directly reflect “real-world” clinical therapeutic outcomes in SCLC patients. In conclusion, we bring forth an established ex vivo CTC expansion system that not only maintains cell viability but is also proficient at conducting drug sensitivity assays capable of predicting therapeutic outcome in SCLC patients—creating access to CTCs for personalized oncology.

## 4. Materials and Methods

### 4.1. Patients and Specimen Collection

This study was approved by the Taipei Medical University Joint Institutional Review Board (N201604036). Patients with SCLC were included in the study and provided written informed consent to participate in this study. For patients enrolled, clinical profiles, including initial cancer staging, medication information, treatment responses, progression-free survival and overall survival, were recorded. Blood samples were collected after patients gave their consent to participate. Multidisciplinary cancer teams in the hospital discussed the treatment plan for cancer patients. The treatment responses were assessed according to the Response Evaluation Criteria in Solid Tumors (RECIST) version 1.1. Patients were followed up at 3-month intervals, and the disease status was documented. Treatment-related toxicity was evaluated by the Common Terminology Criteria for Adverse Events (CTCAE) version 4.03. Contrast-enhanced CT imaging was used to assess tumors and metastatic diseases for different sites.

### 4.2. CTC Enrichment and Expansion

Peripheral blood (7.5 mL) samples were collected in vacated ethylenediaminetetraacetic acid (EDTA) tubes (BD Biosciences, San Jose, CA, USA), and peripheral blood mononuclear cells (PBMCs) were isolated using Ficoll-Paque (Merck, Kenilworth, NJ, USA) and LeucoSep tube (Greiner Bio-One International, Kremsmünster, Austria) method. Blood was added to a LeucoSep tube with Ficoll-Paque Plus and centrifuged to obtain the PBMC fraction. The pellet was resuspended in phosphate-buffered saline (PBS) containing 1% BSA, 2 mM EDTA and CTC enriched by RosetteSep™ CTC Enrichment Cocktail kit (Stem cell technologies, Cambridge, MA, USA). Briefly, the PBMC pellet incubated with antibodies for 20 min at room temperature, followed by the addition of PBS with 2% FBS to the diluted sample. The diluted sample was added on the top of Ficoll-Paque, and after centrifugation, the enriched cells were obtained and suspended in DMEM/F12 medium containing EGF, bFGF, B27 supplement and platelet lysate (Thermo Fisher Scientific, Inc., Waltham, MA, USA). Cells were seeded onto a substrate called binary colloidal crystals (BCCs). One BCC composed of Si and PMMA particles was selected from our BCC library in this study [25]. The medium was replaced in each well every four days. The CTC tumor spheroids were visualized by microscopy. In a parallel study, CTC enumeration by IsoFlux™ system was performed according to the manufacturer’s protocol.

### 4.3. Immunofluorescence Staining

The immunofluorescence staining was used to confirm CTCs, and cells were fixed with paraformaldehyde for 10 min. After being washed with PBS, CTCs were incubated in 0.1% Triton X-100 in PBS for permeabilization. Primary antibodies anti-EpCAM (clone: EpAb3-5, Biomab, Taipei, Taiwan), anti-cytokeratin (clone: AE1/AE3, Thermo Fisher), anti-CD45 (clone: HI30), anti-PDL1 (clone: B7-H1, BioLegend, San Diego, CA, USA), anti-synaptophysin (clone: YE269), anti-TTF1 (clone: EPR5955, Abcam, Cambridge, UK), anti-E-cadherin (clone: 24E10, Cell Signaling, Danvers, MA, USA), anti-N-cadherin (clone: 32/N-Cadherin, BD Bioscience) were used to analyze CTCs and cell nuclei were counterstained by Hoechst 33342. Images were examined and scanned using fluorescence microscopy.

### 4.4. Viability Assay and Drug Screening

A Fluorescence-based viability assay was performed using a LIVE/DEAD^®^ viability/cytotoxicity kit according to the manufacturer’s instructions (Thermo Fisher). Briefly, CTCs were collected and washed with PBS for 5 min. Then, CTCs were incubated with calcein AM and ethidium homodimer (EthD)-1 for 45 min at room temperature. CTCs were washed with PBS, and images were captured by fluorescence microscopy. Viable CTCs were stained green and dead cells were stained red. For drug screening, CTC spheroids were allowed to grow for six weeks, after which spheroids were harvested and seeded in 96-well plates and incubated for 16 h. Cisplatin at 5 μg/mL and 5 μM etoposide (MedChenExpress, Monmouth Junction, NJ, USA) were added to cells and incubated for six days. Cell viability was determined by a CellTiter-Glo^®^ Luminescent Cell Viability Assay (Promega, Madison, WI, USA), in which cells were incubated with Cell-Tilter Glo reagent for 30 min and the luminescence was read on a GloMax^®^ Navigator Microplate Luminometer (Promega). The relative cell viability was calculated as the percentage of cells treated with drugs compared to untreated control cells.

## 5. Conclusions

SCLC is an extremely aggressive cancer characterized by rapid growth, early metastatic spread and reduced responsiveness to therapy. In this study, we successfully established a high-efficiency and reproducible ex vivo CTC expansion system for SCLC. Expanded CTCs from SCLC formed multiple types of spherical tumor structures and expressed SCLC-specific tissue markers. The drug sensitivity assay of expanded CTCs demonstrated that in vitro drug response was capable of predicting clinical therapeutic outcomes. Thus, SCLC patient-derived CTC spheroids may be useful for biomarker development and drug sensitivity testing to provide “real-world” therapeutic prediction response conditions.

## Figures and Tables

**Table 1:** Clinical characteristics of samples used for the expansion of circulating tumor cells (CTCs).

No.	Gender	Age	AJCC Stage	SCLC Stage	Smoking	Successful CTC Expansion	CTC Counts
No. 1	F	62	IV	Extensive	Y	Y	134
No. 2	M	67	IV	Extensive	Y	Y	433
No. 3	M	57	IV	Extensive	Y	Y	16
No. 4	M	61	IV	Extensive	Y	N	277
No. 5	M	71	IV	Extensive	Y	Y	60
No. 6	M	60	IV	Extensive	Y	N	48
No. 7	F	66	IV	Extensive	Y	N	50
No. 8	M	33	IV	Extensive	Y	Y	24
No. 9	M	65	IV	Extensive	Y	Y	15
No. 10	M	67	IV	Extensive	Y	Y	18
No. 11	M	68	IV	Extensive	Y	Y	14
No. 12	F	51	IV	Extensive	Y	Y	15
No. 13	M	87	IV	Extensive	Y	Y	34
No. 14	M	71	IIIB	Limited	Y	Y	12
No. 15	M	43	IV	Extensive	Y	Y	249
No. 16	M	77	IIA	Limited	N	Y	N/A
No. 17	F	60	IV	Extensive	Y	N	190
No. 18	F	89	IB	Limited	N	Y	8
No. 19	F	74	IV	Extensive	N	Y	20
No. 20	F	71	IV	Extensive	Y	Y	130
No. 21	M	79	IIB	Limited	Y	Y	94
No. 22	M	82	IV	Extensive	Y	Y	8

N/A: non-available.
